# CT colonography: can we achieve an adequate bowel preparation without diet restriction?

**DOI:** 10.1007/s00330-023-09471-w

**Published:** 2023-02-18

**Authors:** Marco Rengo, Filippo Tiberia, Simone Vicini, Davide Bellini, Michela Celestre, Gianfranco Trionfera, Andrea Laghi, Iacopo Carbone

**Affiliations:** 1 Department of Medico-Surgical Sciences and Biotechnologies, Academic Diagnostic Imaging Unit, ICOT Hospital, “Sapienza” University of Rome, Via Franco Faggiana, 1668, 04100 Latina, Italy; 2 Department of Radiological, Oncological and Pathological Sciences, Academic Diagnostic Imaging Unit, ICOT Hospital, “Sapienza” University of Rome, Via Franco Faggiana, 1668, 04100 Latina, Italy; 3 Diagnostic Imaging Unit, Valmontone Hospital, Via Dei Lecci, Valmontone, RM Italy; 4 Department of Surgical and Medical Sciences and Translational Medicine, Radiology Unit, Sant’Andrea University Hospital, “Sapienza” University of Rome, Via Di Grottarossa 1035, 00189 Rome, Italy

**Keywords:** Colonography, computed tomographic, Bowel preparation solution, Diet, Patient compliance

## Abstract

**Objective:**

To evaluate if an adequate bowel preparation for CT colonography, can be achieved without diet restriction, using a reduced amount of cathartic agent and fecal tagging. To investigate the influence of patients’ characteristics on bowel preparation and the impact on patients’ compliance.

**Methods:**

In total, 1446 outpatients scheduled for elective CT colonography were prospectively enrolled. All patients had the same bowel preparation based on a reduced amount of cathartic agent (120 g of macrogol in 1.5 l of water) the day before the exam and a fecal tagging agent (60 ml of hyperosmolar oral iodinated agent) the day of the exam. No dietary restrictions were imposed before the exam. The bowel preparation was evaluated using a qualitative and quantitative score. Patients were grouped by age, gender, and presence of diverticula in both scores. Patients’ compliance has been evaluated with a questionnaire after the end of the exam and with a phone-calling interview the day after the exam.

**Results:**

According to the qualitative score, adequate bowel preparation was achieved in 1349 patients (93.29%) and no statistical differences were observed among the subgroups of patients. Quantitative scores demonstrated that colon distension was significantly better in younger patients and without diverticula. A good patients’ compliance was observed and most patients (96.5%) were willing to repeat it.

**Conclusions:**

The lack of diet restriction does not affect the quality of CTC preparation and good patient’s compliance could potentially increase the participation rate in CRC screening programs.

**Key Points:**

• *An adequate quality bowel preparation for CT colonography can be achieved without diet restriction, using a reduced amount of cathartic agent (120 g of macrogol in 1.5 l of water) and fecal tagging (60 ml of hyperosmolar oral iodinated agent).*

• *A bowel preparation based on the combination of a reduced amount of cathartic agent and fecal tagging, without diet restriction, allows obtaining good quality in more than 90% of patients.*

• *The bowel preparation scheme proposed reduces the distress and discomfort experienced by the patients improving adherence to CTC.*

## Introduction

Computed tomography colonography (CTC) is an accurate and minimally invasive method for colonic imaging that has been already approved by the US Preventive Services Task Force, the American Cancer Society, and the European Society of Gastrointestinal Endoscopy (ESGE) together with the European Society of Gastrointestinal and Abdominal Radiology (ESGAR) as an effective test for colorectal cancer (CRC) in average-risk individuals [1–5]. Recently, some studies were conducted investigating the potential role of CTC as a primary screening test by comparing it with other CRC screening methods. The result showed that although the detection rate of CTC was slightly lower, especially if compared to the optical colonoscopy (OC), this may be counterbalanced by the higher CTC participation rate [5–9].

The most frequent reasons given for not adhering to CRC screening programs are related to concern about discomfort experienced during the procedure and about the distress during the days before and after the exam due to bowel preparation [10]. One of the main benefits of CTC, compared to the other colon study investigations, is the possibility of using a reduced bowel preparation as this is usually described by patients as the most annoying part of the exam; moreover, patients report that the discomfort experienced during CTC is less than the tested one during OC [11–14].

According to ESGAR guidelines, the administration of a cathartic agent and fecal tagging with a dietary restriction is mandatory before CTC [15]. Many studies have focused on the cathartic regime but there are few data on the rule of diet restriction [16–19]. Currently, there is no common consensus about the regime of the dietary restriction and guidelines suggest following a low-residual diet period of 24 h or more before the exam [15, 20].

Bellini and co-workers demonstrate that diet restriction can be avoided, since it does not significantly affect the quality of bowel preparation; in addition, the lack of diet-restriction results in better patients’ compliance with the exam [21]. Thus, the primary aim of this study is to confirm these preliminary results, on a larger population. As secondary aims, we investigated the influence of patients’ characteristics on the quality of bowel preparation and the impact of bowel preparation without diet restrictions on patients’ compliance.

## Materials and methods

This non-randomized, single-center, prospective study was conducted according to Good Clinical Practice (GCP)-International Conference on Harmonization (ICH) [22] and approved by the Local Review Board. Informed consent was obtained from all patients.

### Study population

We prospectively enrolled all consecutive outpatients scheduled for elective CT colonography from January 2017 to December 2019.

Inclusion criteria were considered: (a) patients with CRC familiar history or positive fecal occult blood test (FOBT), unwilling to undergo OC; (b) patients presenting with lower bowel symptoms unwilling to undergo OC; (c) patients with a previous incomplete OC.

Exclusion criteria were (a) known allergy to iodinated contrast media; (b) active inflammatory bowel disease; (c) cognitive behavioral deficits (unable to follow instructions); (d) known or suspected pregnancy; (c) patients younger than 18 years old.

### Bowel preparation

**Fig. 1:**
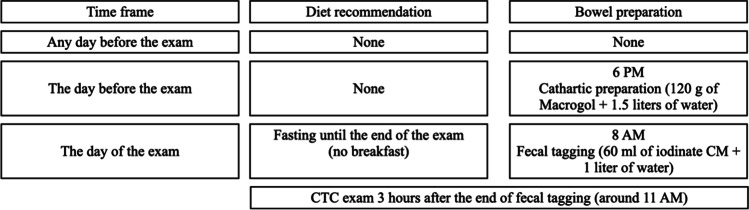
Diet recommendations and bowel preparation scheme

All CTC examinations were acquired in the morning. All patients followed the same bowel preparation as follow. No dietary restrictions were imposed the day before the exam. Patients were instructed to follow their regular diet regimens for breakfast, lunch, and dinner on the day before the exam. The day before the exam patients were asked to intake a dose (120 g) of cathartic agent (Macrogol, ColonPeg®, SANITAS FARMACEUTICI 1931, Italy) in a solution with 1.5 l of water at 6 PM. On the day of the exam, patients were asked to fast, avoiding breakfast. On the day of the exam, at 8 AM, 60 ml of hyperosmolar oral iodinated agent (sodium diatrizoate and meglumine diatrizoate solution, 370 mgI/ml, Gastrografin®, Bayer Pharma) followed by 1 l of water was orally administered to all patients. The CTC exam was performed, for all patients, after a minimum of 3 h or after a clear (limpid liquid) evacuation. The schedule for the bowel preparation as well as for diet recommendations is summarized in Fig. 1.

### CTC acquisition protocol

All CTC examinations were performed using the same MDCT scanner (Aquilion Lightning 80 MDCT; Canon Medical Systems, USA), using the following parameters: 50 mAs, 100 kV, 0.5-mm section thickness, and pitch of 1.388/HP 111.0. Iterative reconstructions were used (AIDR 3D, Canon Medical Systems, USA). Patients were scanned first in a prone and then in a supine position. The scan range for both acquisitions was from the diaphragmatic dome to the proximal third of the legs.

Colon distension was achieved using a rectal lubricated rubber catheter and manual insufflation of room air, performed by a professional nurse until the patient’s maximum tolerance. The quality of colon distension was evaluated on the scanogram and additional air insufflation was considered if necessary before each acquisition. No spasmolytic agents nor intravenous iodinated contrast media were administrated.

The radiation exposure was recorded for each patient collecting the cumulative dose length product (DLP).

### Image analysis

Datasets were anonymized and transferred to a dedicated workstation (Vitrea® Advanced Visualization, CT Colon Analysis. Canon Medical Systems, USA) and analyzed by two independent radiologists with more than 10 years of experience in CTC.

Even though the reconstructed dataset utilized a preset CTC window (window width, 2000 HU; window level, +0 HU), the readers were given the possibility to adapt the window-level settings to their best preference.

The bowel preparation was evaluated using two different scores.

The *qualitative score* was assessed considering three parameters: the quality of fluid tagging, the colon distension, and the complete colon evaluation. All three qualitative parameters were dichotomic (adequate/inadequate) and evaluated considering altogether all colonic segments and both acquisitions.

The quality of fluid tagging was considered adequate if in all colon segments, in one or both acquisitions, the tagged fluids were homogeneous with minimal untagged stools to allow the evaluation of the entire colon wall. If one of these characteristics was not respected the quality of fluid tagging was considered inadequate.

The quality of colon distension was considered adequate if all colon segments, in one or both acquisitions, were sufficiently distended to allow the evaluation of the entire colon wall. If one of these characteristics was not respected the quality of colon distension was considered inadequate.

The evaluation of the colon was considered complete if both fluid tagging and colon distension were adequate and no movement or beam hardening artifacts were observed. If one of these characteristics was not respected, the evaluation of the colon was considered incomplete.

Finally, an overall quality score was calculated. The overall quality was considered adequate if all three parameters were scored as adequate. If one of the three qualitative parameters was scored as inadequate, the overall score was considered inadequate.

**Fig. 2:**
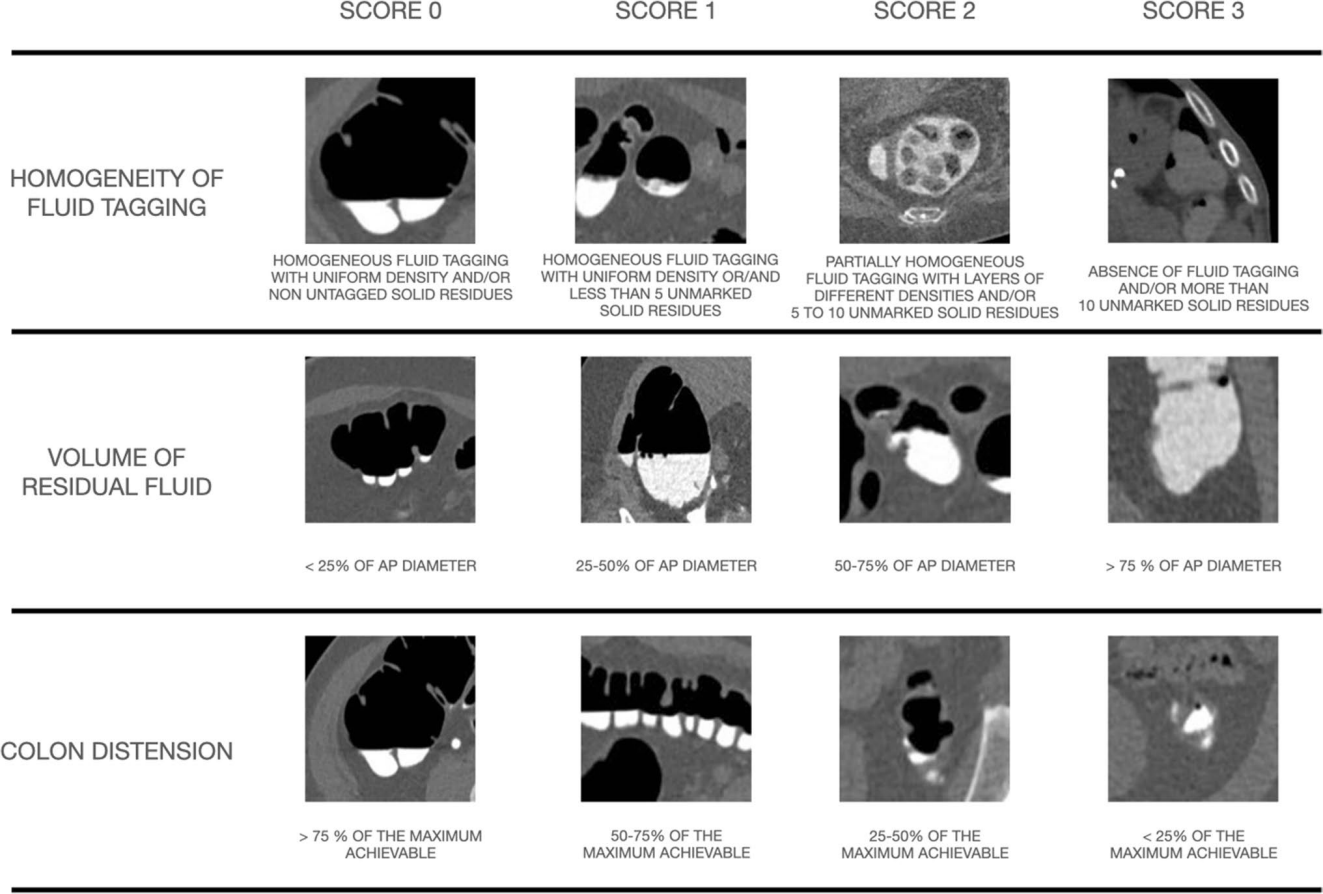
Likert scale used for the quantitative scores

The *quantitative score* was assessed according to a previous method based on a 4-point Likert scale [23]. Three quantitative parameters were assessed: homogeneity of fluid tagging, volume of residual fluids, and colon distension rate. For each parameter a score ranging from 0 to 3 was assigned for each colon segment, dividing the colon into eight segments (caecum, ascending colon, hepatic flexure, transverse colon, splenic flexure, descending colon, sigmoid colon, and rectum) separately for the supine and prone acquisition. The quantitative scores are represented in Fig. 2.

### Patients’ compliance

After the end of the CT acquisition, a questionnaire was administered to all patients. Patients were asked to quantify the discomfort experienced during the entire period including the preparation and the procedure by indicating it on a 100-mm visual analogue scale (VAS). Patients were also asked to quantify the number of evacuations performed from the beginning of the preparation until the exam acquisition.

A phone-calling interview was carried out the day after the exam. Patients were asked to quantify the number of evacuations performed after the exam and the willingness to repeat the procedure.

### Statistical analysis

To evaluate if the proposed scheme allows for achieving adequate quality of bowel preparation, the one used for optical colonoscopy was considered a reference [24]. According to OC guidelines, the minimum standard rate of adequate preparation is 90%.

We investigated the influence of patients’ characteristics on bowel preparation. We divided the population into three groups according to gender (male, female), age (older than 65 years, younger than 65 years), and presence of diverticula. The presence of colonic diverticula was considered if at least 5 diverticula were present in at least one colonic segment.

Differences in quality scores (quality of fluid tagging, colon distension, and complete colon evaluation) among the three groups were calculated using a *χ*^2^ test with Yates correction.

A sample size of 232 patients was required to achieve a statistical power of 95%. This was calculated based on the overall quality score which ranges from 0 to 6. The analysis was performed using dedicated software (G*Power, version 3.1.9.6; University of Dusseldorf, Dusseldorf, Germany) using the *χ*^2^ tests—Goodness-of-fit tests, given an effect size of 0.3, α of 0,05, β of 0,95 and considering the maximum difference of 6 points on the overall quality score.

Differences in quantitative scores (homogeneity of fluid tagging, volume of residual fluids, and colon distension) among the three groups were calculated using the Kolmogorov–Smirnov test.

The quantitative scores were also calculated for each colon segment summing the 3 scores. This analysis was performed for the prone and the supine acquisition separately. Moreover, a third analysis was performed by summing the scores of supine and prone acquisitions (total quality score). Differences were evaluated using the Kruskal–Wallis test for multiple comparisons.

Since there is no evidence of a recommended threshold for quantitative scores to consider the preparation as adequate, we perform a ROC analysis, with the Youden index, to identify this threshold using the overall qualitative score as a reference. In this analysis, we summed all quantitative scores for each segment and for both acquisitions. Thus, the minimum score, corresponding to the best preparation was considered 0 while, the maximum score, corresponding to the worst preparation was 144 (max score for each segment: 3; scores: 3; segments: 8; acquisitions: 2).

The reproducibility of both qualitative and quantitative scores was evaluated by calculating the inter and intra-reader agreement with Interclass Correlation Coefficient (ICC). One of the two radiologists performed all measurements twice for intra-reader agreement. The agreement was interpreted according to the following criteria: *κ* > 0.81: excellent agreement; *κ* = 0.61–0.80: good agreement; *κ* = 0.41–0.60: moderate agreement; *κ* = 0.21–0.40: fair agreement; *κ* < 0.20: poor agreement.

For patient’s discomfort was measured using a 100 mm VAS. Discomfort was considered absent at 0 mm, mild between 1 and 30 mm, moderate between 31 and 59 mm, severe between 60 and 79, very severe between 80 and 99 mm, and worst discomfort possible at 100 mm.

All statistical analysis was carried out using SPSS (Version 25.0.Armonk, NY: IBM Corp.), GraphPad Prism version 7.0 (GraphPad Software, La Jolla, CA, USA), and MedCalc (MedCalc Software® version 12.5, Ostend, Belgium).

All continuous variables were expressed as mean and standard deviation (SD).

A two-tailed *p* < 0.05 was considered statistically significant.

## Results

### Population

We enrolled 1446 consecutive patients (mean age was 62.37 ± 14.1 years; 42% male). In total, 522 patients (36%) were considered at increased risk for CRC (familiar history or positive FOBT) and 220 (15%) underwent CTC after incomplete OC. The average DLP was 180.68 mGy/cm (± 60.09). The characteristics of the study population are summarized in Table 1.

**Table 1:** Demographics and baseline patients’ characteristics

Population	1446
Gender	
Male	605 (42%)
Female	841 (58%)
Age mean ± SD (min–max)	62.37 ± 14.1 (16–90) years
≤ 65	684 (47%)
> 65	762 (53%)
Diverticula	
Presence	614 (43.5%)
Absence	832 (56.5%)
Mean height	170 ± 9 cm
Mean weight	75 ± 13 kg
Mean BMI	26 ± 3 m/kg^2^

### Qualitative score

According to the qualitative score the overall quality of bowel preparation was considered adequate in 1349 patients (93.29%). In detail, an adequate quality of fluid tagging was reached in 1387 patients (95.92%), an adequate colon distension in 1428 patients (98.75%), and a complete colon evaluation were obtained in 1406 patients (97.2%). No significant statistical differences were found in grouping the population according to gender, age, or presence of diverticula. Results are summarized in Table 2.

**Table 2:** Qualitative scores

		Gender			Age			Diverticula		
		Male	Female	*p* value	< 65	≥ 65	*p* value	Yes	No	*p* value
Quality of fluid tagging	Adequate	581 (96%)	806 (95.8%)	0.9602	735 (96.45%)	651 (95.3%)	0.3361	596 (96.5%)	791 (96.6%)	0.3232
	Inadequate	24 (4%)	35 (4.2%)		27 (3.55%)	32 (6.7%)		21 (3.5%)	38 (3.4%)	
Colon distension	Adequate	598 (98.8%)	830 (98.6%)	0.9333	757 (99.3%)	671 (98%)	0.0583	605 (98.05%)	823 (99.28%)	0.0670
	Inadequate	7 (1.2%)	11 (1.4%)		5 (0.7%)	13 (2%)		12 (1.95%)	6 (0.73%)	
Complete colon evaluation	Adequate	585 (96.7%)	821 (97.6%)	0.3689	747 (97.8%)	659 (96.91%)	0.0732	598 (96.91%)	808 (97.6%)	0.6424
	Inadequate	20 (3.3%)	20 (2.4%)		15 (2.2%)	25 (2.29%)		19 (2.29%)	21 (2.4%)	

### Quantitative score

The quantitative scores were compared according to gender, age, or presence of diverticula. The only significant statistical difference was observed for colon distension. Colon distension was lower in patients with diverticula (*p* = 0.006) and in older patients (*p* = 0.0001). Both homogeneity of fluid tagging and volume of residual fluids were not influenced by any of the patient’s characteristics. Results are summarized in Table 3.

**Table 3:** Quantitative scores

	Gender			Age			Diverticula		
	Male	Female	*p* value	< 65	≥ 65	*p* value	Presence	Absence	*p* value
Homogeneity of fluid tagging	0.13 ± 0.43	0.11 ± 0.47	0.1481	0.10 ± 0.42	0.14 ± 0.49	0.9989	0.10 ± 0.40	0.14 ± 0.49	0.9999
Volume of residual fluids	0.07 ± 0.22	0.07 ± 0.25	0.9971	0.05 ± 0.19	0.08 ± 0.28	0.7499	0.05 ± 0.19	0.08 ± 0.27	0.897
Colon distension	0.07 ± 0.21	0.07 ± 0.2	0.9966	0.05 ± 0.19	0.10 ± 0.27	**0.006**	0.11 ± 0.30	0.04 ± 0.15	**0.0001**

The analysis performed per segment revealed that the qualitative score was significantly higher for the sigmoid colon, compared to all the other segments, in the prone and supine acquisition as well as in the total qualitative score. Results are summarized in Table 4.

**Table 4:** Quantitative scores. Per segment analysis

	PRONE			SUPINE			TOTAL		
	SCORE 0	MEAN	SD	SCORE 0	MEAN	SD	SCORE 0	MEAN	SD
RECTUM	1334 (92.25%)	0.15	0.64	1345 (93.024%)	0.17	0.92	1332 (92.13%)	0.32	1.45
SIGMOID	1065 (73.2%)	0.42	0.88	1067 (73.77%)	0.41	0.88	1054 (72.86%)	0.83	1.76
DESCENDING	1159 (80.16%)	0.33	0.88	1166 (80.62%)	0.32	0.81	1153 (79.70%)	0.65	1.64
SPLENIC FLEX	1268 (87.69%)	0.22	0.73	1276 (88.26%)	0.22	0.73	1266 (87.57%)	0.44	1.46
TRASVERSUM	1275 (88.14%)	0.21	0.72	1279 (88.48%)	0.21	0.72	1271 (87.91%)	0.42	1.43
HEPATIC FLEX	1289 (89.17%)	0.21	0.75	1294 (89.51%)	0.21	0.75	1288 (89.5%)	0.43	1.5
ASCENDING	1250 (86.43%)	0.27	0.81	1246 (86.20%)	0.27	0.81	1243 (85.97%)	0.54	1.61
CIECUM	1240 (85.75%)	0.28	0.82	1242 (85.86%)	0.28	0.82	1238 (85.63%)	0.56	1.64

**Fig. 3:**
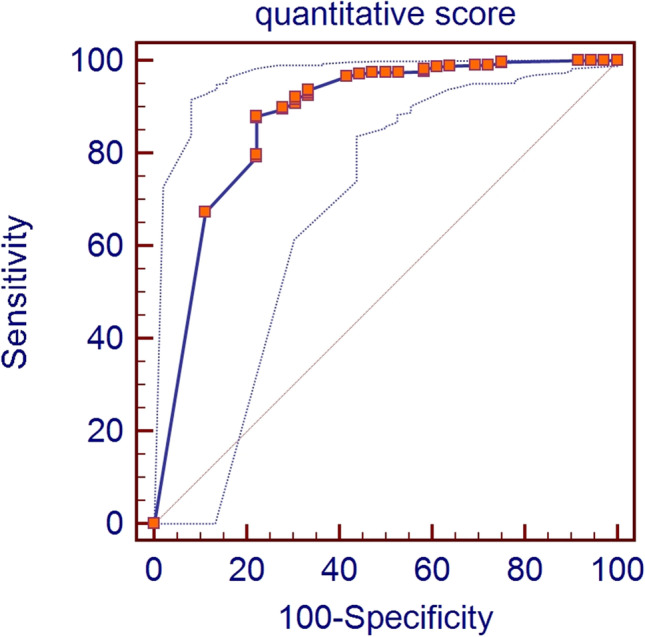
The figure shows the area under the curve (AUC) of the receiver operating characteristic (ROC) curve analysis with 95% confidence limits (AUC = 0.875 and CI: 0.842–0.903)

As defined by the protocol, the total quantitative score ranges from 0 to 144, considering 8 colon segments in both supine and prone acquisition. According to the ROC analysis (Fig. 3) a cumulative quantitative score equal or inferior to 5/144 should be considered the threshold to define the quality of bowel preparation as adequate. Using this threshold (≤ 5) the AUC was 0.875 (95% CI: 0.842–0.903), SE 0.88 (95% CI: 0.84–0.91), and SP 0.78 (95% CI: 0.61–0.90). The average total quantitative score of the study population was 4.16 (± 10.57).

### Patients’ compliance

The questionnaire administered immediately after the exam reported that 78.8% of patients had less than 5 evacuations before the exam and the average discomfort, during the procedure was 26.3/100 (± 11.3). The phone-calling interview, conducted one day after the exam, reported that 79.8% of patients had less than 5 evacuations after the exam and the majority of these (96.5%) were willing to repeat CTC exam (Table 5).

**Table 5:** Patients’ compliance

Number of evacuations	
> 5	21.3%
≤ 5	78.7%
Before the exam	20.7%
After the exam	79.3%
Mean pain	26.3/100 (± 11.3)
Willing to repeat the exam	
Yes	96.3%
No	3.7%

### Reproducibility

The two scores showed good or excellent agreement for both inter- and intra-reader evaluation. The qualitative scores showed an intra-reader agreement of ICC = 0.93 (95% CI: 0.86–1.00) and an inter-reader agreement of ICC = 0.91 (95% CI: 0.81–0.98). The quantitative scores showed an intra-reader agreement of *κ* = 0.82 (95% CI: 0.59–0.94) and an inter-reader agreement of *κ* = 0.79 (95% CI: 0.53–0.93).

## Discussion

The primary outcome of this study demonstrated that adequate bowel preparation for CT colonography can be achieved without diet restrictions, using a reduced amount of cathartic agent and fecal tagging. In this setting, which might be considered a “real-world scenario,” an adequate bowel preparation was achieved in 93.29% of the study population. This result is consistent with what we considered as the reference standard, the ESGE guidelines for OC, which requires that bowel preparation should be adequate for at least 90% of the population [24]. These results can also be considered as a confirmation of the outcomes of the study of Bellini et al [21] that previously demonstrated the absence of significant differences between bowel preparations with and without diet restrictions using cathartic and fecal tagging regimens, similar to the ones used in the present study.

One of the secondary outcomes of this study was to evaluate the influence of patients’ characteristics, like age, gender, and the presence of diverticula on the quality of bowel preparation. The qualitative scores (quality of fluid tagging, colon distension, and complete colon evaluation) were not influenced by patients’ gender and age nor by the presence of colonic diverticula. However, the quantitative scores (homogeneity of fluid tagging, volume of residual fluids, and colon distension) demonstrated some differences among the three subgroups. The quantitative analysis demonstrated inferior bowel distension in older patients and in those with colonic diverticula. These outcomes are in line with previous studies demonstrating that bowel distension is reduced in older patients [25] or in patients with colonic diverticula [26, 27]. Moreover, the quantitative score revealed a significantly lower quality of the preparation for the sigmoid colon, which represents the most frequent location of the colonic diverticula.

The quantitative score, first introduced by Taylor et al [23], can be considered an excellent method to compare different bowel preparation regimens as confirmed by its extensive use in many previous studies [17, 21, 28–30]. However, this method cannot be used to determine if the overall bowel preparation is adequate or not. Since several bowel preparation quality scores have been validated for OC [31], we correlated Taylor’s quantitative score to the qualitative score used in this study in an attempt to identify a threshold to discriminate between adequate or inadequate preparation. Our data suggests that a threshold of ≤ 5/144 can be considered sufficiently accurate (AUC was 0.875; 95% CI: 0.842–0.903) to identify adequate bowel preparation. This threshold might be considered restrictive, but we should consider that, according to the qualitative score used, only datasets with perfect distension and tagging were scored as adequate. Moreover, the average quantitative score of our study population was below this threshold (4.16 ± 10.57). To the best of our knowledge, no other studies tried to identify a quality score for bowel preparation in CTC.

The last outcome of this study was to evaluate the effect of the proposed bowel preparation scheme on patients’ compliance. The average discomfort reported, experienced during the entire period including the preparation and the procedure, was mild (26.3/100 ± 11.3 on the VAS). Moreover, most patients (96.5%), during the follow-up interview, declared their will to repeat the exam. We believe that these results have been influenced by the simpleness of the preparation and by the minimal effect on the daily routine the day before the exam. The proposed preparation can be considered simple since no diet restrictions were imposed and the only indication was to intake, the day before the exam, a reduced amount of cathartic agent in the afternoon, at 6 PM, when patients are usually at home after work. The effect of the cathartic agent can be considered moderate, since most patients (78.8%) declared less than 5 evacuations before the exam. The cathartic effect of the hyperosmolar oral iodinated agent, used for fecal tagging, should be considered since most of the patients declared post-exam evacuations. However, also the number of post-exam evacuations was limited (< 5) in the majority of the population (79.8%). These results are in line with previous publications [16–19] investigating the effect on patients’ comfort and diagnostic quality of different cathartic regimens for CTC, which confirmed the superiority of reduced bowel preparation. Moreover, the distress due to bowel preparation has been demonstrated to be one of the main reasons for not adhering to CRC screening [10], and the reduced preparation for CTC has been demonstrated to provide a higher adherence rate confirming its potential role as a primary screening test [9].

Or study has some limitations. We did not directly assess the impact of the proposed scheme for bowel preparation on diagnostic accuracy. However, diagnostic accuracy is strongly influenced by the quality of bowel preparation thus we can assume that adequate preparation should not negatively influence diagnostic accuracy. The bowel distension was performed manually using room air. The use of an automatic CO2 pump may result in better colon distension. Also, the use of spasmolytic agents may improve colon distension. In our population, we did not use spasmolytic agents nor CO_2_ insufflators; however, colon distension resulted in adequate 98.75% of the study population. Finally, we did not investigate the effect of this preparation scheme on the same day OC. However, the volume of residual fluids in this population was small, as demonstrated by the corresponding quantitative score, thus, we can assume that residual fluids may be easily aspirated during endoscopy.

In conclusion, the results of this study, obtained in a real-world scenario, demonstrated that adequate bowel preparation can be achieved in most patients avoiding diet restrictions and that this bowel preparation scheme has been well tolerated by the majority of the study population. These two outcomes may further improve adherence to CTC CRC screening programs.

## Abbreviations

CRC: Colon-rectal cancer
CTC: Computed tomography colonography
ESGAR: European Society of Gastrointestinal and Abdominal Radiology
ESGE: European Society of gastrointestinal Endoscopy
FOBT: Fecal occult blood test
OC: Optical colonoscopy
VAS: Visual analogue scale

## Declarations

### Guarantor

The scientific guarantor of this publication is Dr. Marco Rengo.

### Statistics and biometry

No complex statistical methods were necessary for this paper.

### Methodology

• prospective

• diagnostic study

• performed at one institution
